# Small-Scale Perfusion Bioreactor of Red Blood Cells for Dynamic Studies of Cellular Pathways: Proof-of-Concept

**DOI:** 10.3389/fmolb.2016.00011

**Published:** 2016-03-30

**Authors:** Michel Prudent, Frédéric Stauber, Alexis Rapin, Sonia Hallen, Nicole Pham, Mélanie Abonnenc, Laure Marvin, Bertrand Rochat, Jean-Daniel Tissot, Niels Lion

**Affiliations:** ^1^Laboratoire de Recherche sur les Produits Sanguins, Recherche et Développement, Transfusion Interrégionale CRSEpalinges, Switzerland; ^2^Quantitative Mass Spectrometry Facility, Centre Hospitalier Universitaire Vaudois (CHUV)Lausanne, Switzerland

**Keywords:** bioreactor, erythrocyte, metabolism, storage, transfusion

## Abstract

To date, the development of bioreactors for the study of red blood cells (RBCs, daily transfused in the case of disease or hemorrhage) has focused on hematopoietic stem cells. Despite the fact that mature RBCs are enucleated and do not expand, they possess complex cellular and metabolic pathways, as well as post-translation modification signaling and gas-exchange regulation. In order to dynamically study the behavior of RBCs and their signaling pathways under various conditions, a small-scale perfusion bioreactor has been developed. The most advanced design developed here consists of a fluidized bed of 7.6 mL containing 3·10^9^ cells and perfused at 8.5 μL/min. Mimicking RBC storage conditions in transfusion medicine, as a proof-of-concept, we investigated the *ex vivo* aging of RBCs under both aerobic and anaerobic conditions. Hence, RBCs stored in saline-adenine-glucose-mannitol (SAGM) were injected in parallel into two bioreactors and perfused with a modified SAGM solution over 14 days at room temperature under air or argon. The formation of a fluidized bed enabled easy sampling of the extracellular medium over the storage period used for the quantitation of glucose consumption and lactate production. Hemolysis and microvesiculation increased during aging and were reduced under anaerobic (argon) conditions, which is consistent with previously reported findings. Glucose and lactate levels showed expected trends, i.e., decreased and increased during the 2-week period, respectively; whereas extracellular glucose consumption was higher under aerobic conditions. Metabolomics showed depletion of glycolsis and pentose phosphate pathway metabolites, and an accumulation of purine metabolite end-products. This novel approach, which takes advantage of a fluidized bed of cells in comparison to traditional closed bags or tubes, does not require agitation and limit shear stress, and constantly segragates extracellular medium from RBCs. It thus gives access to several difficult-to-obtain on- and off-line parameters in the extracellular medium. This dynamic bioreactor system does not only allow us to probe the behavior of RBCs under different storage conditions, but it also could be a powerful tool to study physiological or pathological RBCs exposed to various conditions and stimuli.

## Introduction

Red blood cells (RBCs) are specific cells that are devoid of nuclei and mitochondria and are not able to replicate in their mature state (erythrocytes). They are essential, and in case of hemorrhage or disease, they can be transfused from stored erythrocyte concentrates (ECs). In several recent clinical studies in cardiac surgery and intensive care unit patients, the quality of ECs has been questioned (Koch et al., 2008; Lacroix et al., 2015; Steiner et al., 2015), as different outcomes were observed, feeding the debate (Benjamin and Dodd, 2008; McCullough, 2013; Zimring, 2013; van de Watering, 2013a,b, 2014; Prudent et al., 2015). A large controversy has thus emerged in the transfusion medicine community: not only long-term-stored ECs are presented as biomedical products of lesser efficiency, but also tend to be viewed as harmful therapeutic interventions (Hod et al., 2011; Hod and Spitalnik, 2012; Rubin et al., 2013; Danesh et al., 2014).

During storage, RBCs suffer from storage lesions (Tinmouth and Chin-Yee, 2001; Hess and Greenwalt, 2002; Prudent et al., 2011; Flatt et al., 2014; D'Alessandro et al., 2015), i.e., molecular and cellular modifications of RBCs during blood banking. Firstly, *in vitro* studies have shown the increase of hemolysis (depending on the additive solutions, AS, and the preparation processes) and accumulation of microvesicles (MVs) (Rubin et al., 2008; Sparrow et al., 2014), the loss of metabolic modulation (e.g., adenosine triphosphate, ATP, and 2,3-bisphosphoglycerate, 2,3-DPG, depletion) and pH lowering (Hess et al., 2000; Bennett-Guerrero et al., 2007; Sparrow et al., 2014). Secondly, at the protein level these modifications include the aggregation and degradation of the band 3 protein generating the formation of neoantigens (Bosman et al., 2008), and alteration of proteins (D'Amici et al., 2007; Bosman et al., 2008; Antonelou et al., 2010; Rinalducci et al., 2011; Delobel et al., 2012, 2016; Pallotta et al., 2015). The metabolism plays a central role regulating several ATP-dependent enzymes, antioxidant defenses and other cellular functions. For instance, band 3 complexes regulate the RBC metabolism (Chu et al., 2008; Lewis et al., 2009) and the gas-exchange (Bruce et al., 2003) required to deliver O_2_ to tissues and organs. Metabolomic studies have reported a decrease in glycolysis rate and the accumulation of lactate but some differences were observed in other pathways such as the pentose phosphate pathway (PPP) (Nishino et al., 2009; Gevi et al., 2012; Paglia et al., 2014; Roback et al., 2014). This metabolomic approach was also applied to the study of new storage conditions, such as anaerobic conditions (D'Alessandro et al., 2013) or new AS compositions (Nishino et al., 2013). Even though RBCs are able to counterbalance the lesions during cold storage, they are not able to totally stop the reversible degradation that is the prelude to irreversible lesions. Current knowledge of storage lesions and RBC aging mechanisms has to expand to improve RBC storage conditions and to better understand the links to adverse clinical outcomes that may participate in pro-thrombotic events (Jy et al., 2013; Rubin et al., 2013) and inflammatory effects (Hod et al., 2011; Hod and Spitalnik, 2012; Danesh et al., 2014).

Myriad solutions can be considered to reduce storage lesions. The addition of antioxidants (such glutathione, GSH, or ascorbic acid) or precursor of the GSH synthesis prevents oxidative stress (ascorbic acid and N-acetylcysteine) (Dumaswala et al., 2000; Pallotta et al., 2014), but decrease the energy metabolism (Pallotta et al., 2014). Another approach is the storage under anaerobic conditions. The principle of anaerobic storage is quite trivial and consists of replacing O_2_ by an inert gas such as helium or argon. Zolla and colleagues stored RBCs under helium to protect the cells against oxidative stress and observed reduced protein degradation (D'Amici et al., 2007). To ensure the complete depletion, Yoshida and colleagues developed a system where, first, the O_2_ was depleted by argon, and second, the ECs were placed in an anaerobic canister filled with Ar/H_2_ (9/1). Importantly, this canister contained a palladium catalyst that guaranteed depletion during storage (Yoshida et al., 2007; Dumont et al., 2009; Yoshida and Shevkoplyas, 2010). Using this system, they significantly improved the ATP level, but not 2,3-DPG, during RBC storage (2-fold after 63 days) and could push the storage limit up to 9 weeks (vs. 6 weeks for storage in permeable bags containing saline-adenine-glucose-mannitol, SAGM) (Yoshida et al., 2007). They also observed a decrease in microvesiculation, demonstrating a benefit to RBC membranes. Regarding metabolomics, glycolysis was enhanced (Kinoshita et al., 2007; Lewis et al., 2009; D'Alessandro and Zolla, 2013). However, under these conditions, the shift to the PPP was partially blocked, thus reducing the glutathione-based defenses (less glutathione and increased level of oxidized glutathione) and attenuating the effect of anaerobic storage (Dumont et al., 2016). Interestingly, the effect of anaerobic storage on carbon monoxide-exposed RBCs on ATP was suppressed, which is well in phase with the Kinoshita and colleagues' work (Kinoshita et al., 2007). These results are in agreement with the known regulation of O_2_-dependent glycolysis (Chu et al., 2008). While intriguing, the aforementioned studies have worked directly on ECs and have required sample preparation prior to analyses.

Research on ECs and RBC aging has long been empirical in transfusion medicine where blood bags or freshly withdrawn blood are used. Solid *in vitro* in addition to *in vivo* data are required to understand the lesions and to target development (Hess, 2006; Prudent et al., 2015). There is a clear unmet need for a dynamic bioreactor which requires fewer samples and less biological sampling, whereas the main developments were dedicated to stirred, fixed-bed or hollow fibers bioreactors for hematopoietic stem cells (Collins et al., 1998; Liu et al., 2006; Timmins et al., 2011; Housler et al., 2012; Ratcliffe et al., 2012). The only published system of mature RBC bioreactor is a closed stirred-tank reactor, enabling gas regulation (Deonikar and Kavdia, 2010). However, numerous solutions are available to segregate cells to a confined space for the purpose of studying their behavior (Butler, 2005; Zhong, 2010). Jolicoeur and colleagues have designed small-scale bioreactors for the culture of plant and mammalian cells (Gmati et al., 2005; Ben-Tchavtchavadze et al., 2010). A similar approach was applied here to develop a small-scale bioreactor of RBCs, taking into account the size and deformability of these cells. The aims of the proposed system were to (i) design a small bioreactor capable of segregating RBCs from medium, providing nutrients, allowing sampling and/or on-line monitoring in the supernatant; and (ii) to study RBC aging and different storage conditions. In future, this device could be a valuable tool to study cellular pathways under various conditions.

## Materials and methods

### Chemicals

NaCl, adenine and mannitol were purchased from Sigma-Aldrich (Steinheim, Germany); ethanol 70% from Fluka analytical (Buchs, Switzerland); and glucose from Merck (Darmstadt, Germany). Physiological 0.9% NaCl was purchased from B. Braun Medical (Sempach, Switzerland) and PBS 10x from Laboratorium Dr. Bichsel (Interlaken, Switzerland). H_2_O used for each solution was deionized water at 18.2 MΩ·cm.

Modified SAGM (mSAGM) was prepared as follows: 150 mM NaCl, 1.2 M adenine, 4.5 mM glucose, and 30 mM mannitol, and the solution was filtered at 0.22 μm and stored at 4°C.

### Preparation of erythrocyte concentrates

ECs were prepared from whole blood donations of 450 ± 50 mL in citrate-phosphate-dextrose anticoagulant and component filtered (Delobel et al., 2012). ECs were stored at 4°C in SAGM at a hematocrit of 0.6 ± 0.1 v/v. ECs that did not meet the quality criteria for blood transfusion, e.g., low hemoglobin content or a slightly too small volume, were used under the signed consent of blood donors. No research on genetic material was carried out. Therefore, no specific ethical processing was required and these samples were used in agreement with the local legislation.

### Small-scale perfusion bioreactor

#### Design

**Figure 1:**
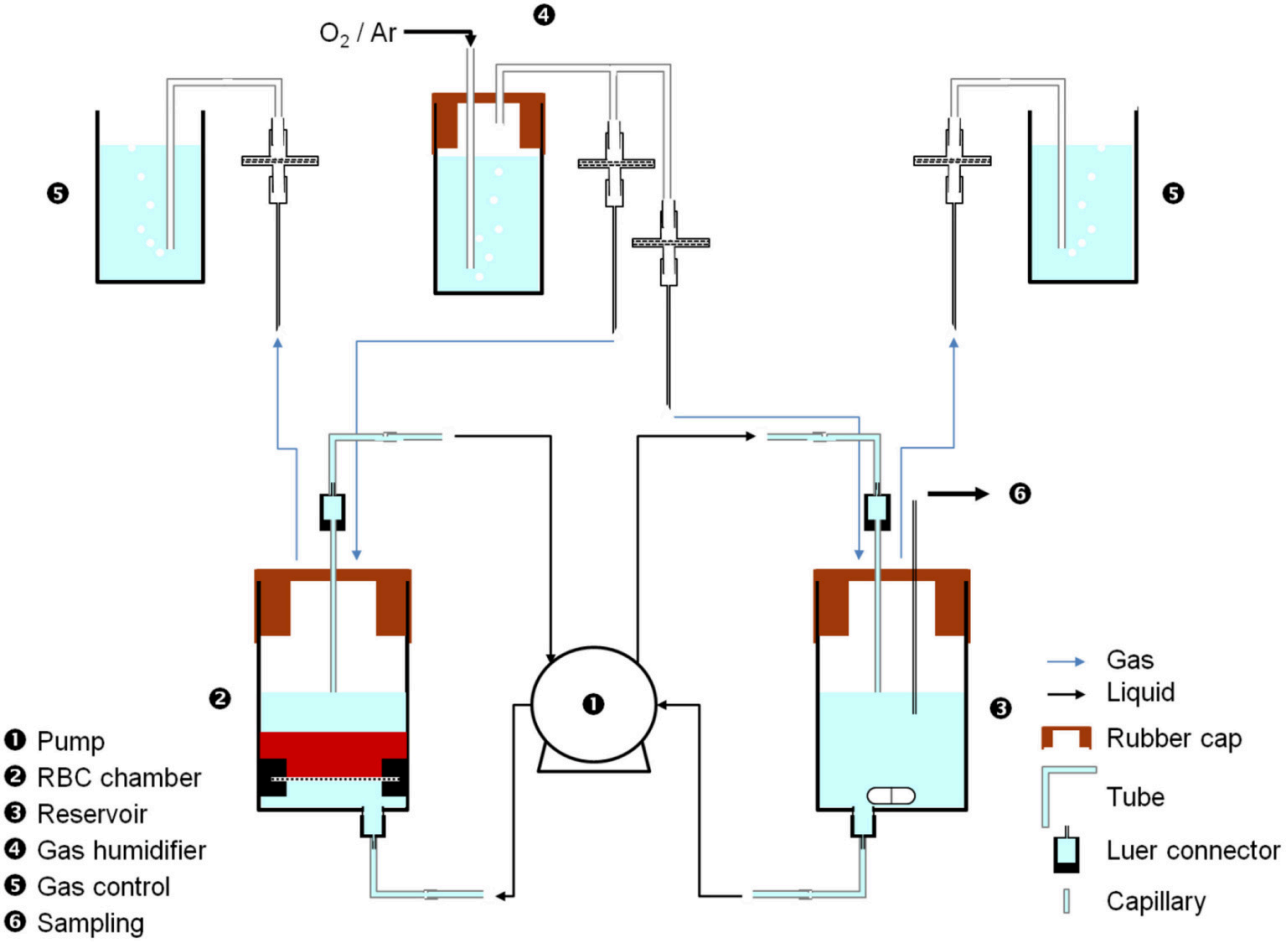
**Small-scale perfusion bioreactor of RBCs**. Medium (mSAGM) was pumped (➊) at 8.5 μL/min from the reservoir (➌) to the chamber (➋) to fluidize RBCs and then pumped back to the reservoir. Gas (air or argon) was supplied to both the chamber and the reservoir, through a humidifier (➍). Sampling was done in the reservoir using a sterile needle through the rubber cap (➍). pH and K^+^ probes can be easily fitted to the reservoir.

Several designs were considered to properly confine RBCs (see Supplementary Figure 1) and the following represents the most advanced design. Each bioreactor includes (see Figure 1) a chamber (20 mL-syringe barrel cut at 15 mL-grad. line, Omnifix, B-Braun, ➋), with qualitative paper filter at the bottom (held by two 20 mL-syringe plunger half rings, Omnifix, B-Braun) to ensure homogenous flow and retain cells before the flow was switched on, a rubber cap (Suba Seal Septa 37, Aldrich), a capillary and luer connectors (BioRad) to interface with the tubing; a reservoir (20 mL-syringe barrel cut at 8 mL-graduation line, Omnifix, B-Braun, ➌), with a stirring magnet to ensure medium homogeneity, a rubber cap, and luer connectors to interface with the tubing. The flow rate was applied using a 4-channel peristaltic pump (Reglo Digital MS-4/8, Ismatec, ➊) capable of running two bioreactors in parallel (2 channels/bioreactor, 1 from the chamber to the reservoir and 1 from the reservoir to the chamber) using tubing for carrying bioreactor medium (Tygon R3607, Saint Gobain Performance Plastics). Different gases (compressed air 2.2 and argon 5.0, Messer, Switzerland) filled the chamber and the reservoir through gas tubing (Tygon ID = 1.6 mm, Saint Gobain Performance Plastics), with filters (25 mm syringe filter, 0.2 μm polyethersulfone membrane, VWR), syringe needles (Neolus, 1.2 × 40 mm, Terumo) as interface tubing-chamber/reservoir, luer and 0.8 mm T-connectors (BioRad). H_2_O-containing gas humidifier chambers (➍) were added to avoid drying the system and a gas evacuation and control chambers (20 mL syringe, B-Braun, ➎), with open vents and filled with H_2_O to check correct gas circulation (constant bubbling, ~2–3 bubbles/s) in every compartment.

#### Bioreactor storage experiments

Two bioreactors were run in parallel under a specific gas condition (air 2.2 or argon 5.0, i.e., aerobic or anaerobic conditions, respectively) during 2 weeks. First, each system was filled with 7 mL of mSAGM solution (5 mL in chambers, 2 mL in reservoirs) and 0.6 mL from an EC was gently loaded into each chamber with a 1 mL syringe (Omnifix, B-Braun). Then, the gas supplies were opened, and the magnetic stirrer and the peristaltic pump turned on. The pump ensured a constant low-flow (8.5 μL/min) medium circulation between the chambers and the reservoirs, and the maintenance of the RBCs fluidized bed with the resulting bottom-up medium flow into the chambers. Antibiotics (penicillin and streptomycin) were also added (see “Discussion”). After 14 days, the system was stopped. Before and after each experiment, the whole system was cleaned out with soap in H_2_0, ethanol 70%, and finally rinsed with H_2_0.

The bioreactor storage experiments were repeated three times on ECs (at 4, 6, and 7 days of storage) originating from three different donors.

#### Sampling

Three mL of an EC was aseptically withdrawn at day 0. Two samples of 0.6 mL from it were loaded in the bioreactors, 15 μL were used for MV counting and 50 μL for hemolysis measurement. Then, the supernatant was collected after centrifugation at 2000 g, 10 min, 4°C and stored at −28°C for hemolysis measurements. RBCs were washed two times in 0.9%, NaCl and stored at −28°C (aliquot of 100 μL of packed RBCs) for intracellular metabolite quantification.

At 2–3 days intervals, a 20 μL sample was removed from each reservoir (➏) using a 1- mL sterile syringe and a sterile needle, and stored at −28°C for further testing (extracellular glucose and lactate concentrations).

At the end of the experiment, the whole content of the bioreactors (medium and cells) was removed and pooled. As day 0 sample, 15 μL of these bioreactor total samples were used for MV counting and 50 μL for hemolysis measurement. RBCs and supernanants were prepared and stored as before.

### Hemolysis, MVs and metabolite analyses

The hemolysis rate was measured on pre-/post-bioreactor samples. Hemoglobin concentration ([Hb]_total_) and hematocrit were obtained with an automated hematology analyzer (KX-21N, Sysmex) from 50 μL of EC and the free hemoglobin concentration in the supernatant ([Hb]_*supernatant*_) was determined by the Harboe method by measuring the absorbance at 415, 380, and 450 nm with a spectrophotometer (UltroSpec 1000, Pharmacia Biotech) (Harboe, 1959) as follows:

(1) $$\begin{array}{r} {\left[Hb\right]}_{supernatant}=\frac{\begin{array}{c} \left(167.2A_{415}-83.6\left(A_{380}+A_{450}\right)\right).\\ dilution\;factor \end{array}}{1000} \end{array}$$

where A stands for the absorbance at different wavelength.

The hemolysis rate in % was obtained with the following equation:

(2) $$\begin{array}{l} Hemolysis=\frac{{\left[Hb\right]}_{supernantant}\cdot \left(100-hematocrit\right)}{{\left[Hb\right]}_{total}} \end{array}$$

The MV concentration was quantified by flow cytometry in pre-/post-bioreactor samples (Delobel et al., 2012). Five micro liter of samples were incubated in triplicate with 5 μL FITC mouse anti-human CD47 antibody and incubated 20 min at room temperature (RT) on a roller. Before analysis, 5 μL of labeled MVs were dissolved in 400 μL of NaCl 0.9% into BD Trucount tubes that contained a precise number of three-time labeled microbeads for quantification.

Extracellular glucose and lactate concentrations were determined from 10 and 4 μL of reservoir samples, respectively, using colorimetric assays according to manufacture protocols (Biochain glucose assay kit, Z5030025, Biochain; Biovision lactate colorimetric assay II, K627-100, Biosion). Absorbances were recorded on a spectrophotometer (Reader 2001, Anthos Labtec Instruments) and the concentrations were calculated from metabolite calibration curves.

### Metabolomics

Intracellular metabolites were extracted from 100 μL pre-/post-storage RBCs samples using a methanol extraction protocol. One hundred microliters of ice-thawed RBCs were mixed with 300 μL of MeOH and sonicated 10 min at RT. Three hundred microliters of the supernatants were transferred to a 1.5 mL tube after centrifugation at 20,817 g, 12 min, 4°C. Metabolite extracts were dried under N_2_ flux for 1 h at RT. Dried extracts were dissolved in 100 μL of ACN/H_2_O 50/50 (v/v), vortexed, sonicated for 10 min and stored on ice for 20 min. After a final centrifugation (as before), 80 μL of solution was transfer to an injection vial.

Four microliters of each sample were injected in triplicates on an LTQ HPLC system (Thermo Scientific, Germany) with a well-plate autosampler thermostated at 4°C (HTS PAL system, CTS Analytics, CH), Rheos pumps (Flux instruments, Switzerland) and a thermostated column compartment. Chromatographic separations were achieved onto a ZIC-pHILIC column (100 mm × 2.1 mm, 5 μm—SeQuant®, Germany) at a column temperature of 40°C. Chromatography conditions are summarized in Table 1 (the buffer was made of ammonium carbonate at 100 mM pH 9.2). High resolution mass spectrometry (MS) analysis was carried out on an Exactive Plus™ MS (Thermo Scientific, Germany) equipped with an electrospray ionization source (H-ESI-II). Mass spectra for metabolite extracted samples were acquired in negative ionization mode. Instrument calibration was performed externally every month with a Pierce® calibration solution (Thermo Scientific, Germany).

**Table 1:** **LC gradient conditions**.

**#**	**Time [min]**	**H_2_O [%]**	**ACN [%]**	**Buffer [%]**	**Flow rate [μL/min]**
0	0	0	95	5	300
1	0.5	0	95	5	300
2	20	25	70	5	300
3	24	45	50	5	300
4	26	75	20	5	300
5	29	75	20	5	300
6	29.1	0	95	5	400
7	33.9	0	95	5	400
8	34	0	95	5	50
9	—	100	0	0	50

Xcalibur software (Thermo Scientific, Germany) was used for data treatment. The mass extraction window was performed with a range of ± 5 ppm and the peak integration parameters were 200 for the baseline window, 10 for the area noise factor and 50 for the peak noise factor. The progenesis-QI software (Non-linear Dynamics Ltd, UK) was used to perform principal component analysis (PCA) with the following parameters: MS profile data, negative; peak picking: automatic sensitivity, minimum peak width of 0.05 min, normalized to all compounds; identification: home-made database.

### Statistical analyses

*t*-test analyses were performed between hemolysis, MV count, lactate and glucose levels in air or argon bioreactors using the software R version 3.0.1 (The R foundation for Statistical Computing). *p*-values lower than 0.05 were considered as significant.

Metabolomic data were analyzed using the progenesis-QI software as described in Section Metabolomics *p*-values lower than 0.05 were considered as significant.

## Results

### Bioreactor conceptualization

Several designs of continuous reactors were investigated, such as open or closed perfusion reactors using filters and fluidized-bed reactors (see Supplementary Figure 1 for the tested designs). Because of the RBC size (around 7 μm) and their ability to pass through small pores of a few microns, the most suitable design was a perfusion bioreactor with the fluidization of RBCs. The flow rate was determined by achieving a distinct and flat layer of RBCs, at 8.5 μL/min. Below this flow rate, the bed collapsed with an accumulation of RBC in the input tube; and above, the convection front continuously increased, where RBCs reached the outlet and recirculated within the whole system. Recirculation of RBCs generated hemolysis (0.7% after 8 passages through the pump and 3.5% after 100 passages using 6 and 7-day old ECs with an initial hemolysis of 0.07 and 0.08%, respectively).

Using an open system was not possible using the AS because the low initial amount of plasma in the EC has been shown necessary to maintain the cell integrity. Indeed, a clear hemolysis (supernatant became clear red, a sign of hemolysis) was observed at day 2 in open systems (inlet reservoir containing SAGM and outlet to the waste) that was explained by washing the RBCs with fresh AS. The remaining plasma inside the ECs was required to limit hemolysis and it was observed that albumin levels as low as 5 g/L were sufficient to prevent RBC lysis (0.4% after 7 days at RT in the presence of BSA at 5 g/L vs. 1.2% without BSA) in AS (see Supplementary Materials and Supplementary Figure 2).

Therefore, the optimal design is illustrated in Figure 1, a small-scale fluidized-bed bioreactor that contained 2.8·10^9^ RBCs in a total volume of 7.6 mL of mSAGM. The system was stable for at least 2 weeks but could be maintained for longer periods of time (as at 4°C for instance).

### Hemolysis, MVs and extracellular metabolites

**Figure 2:**
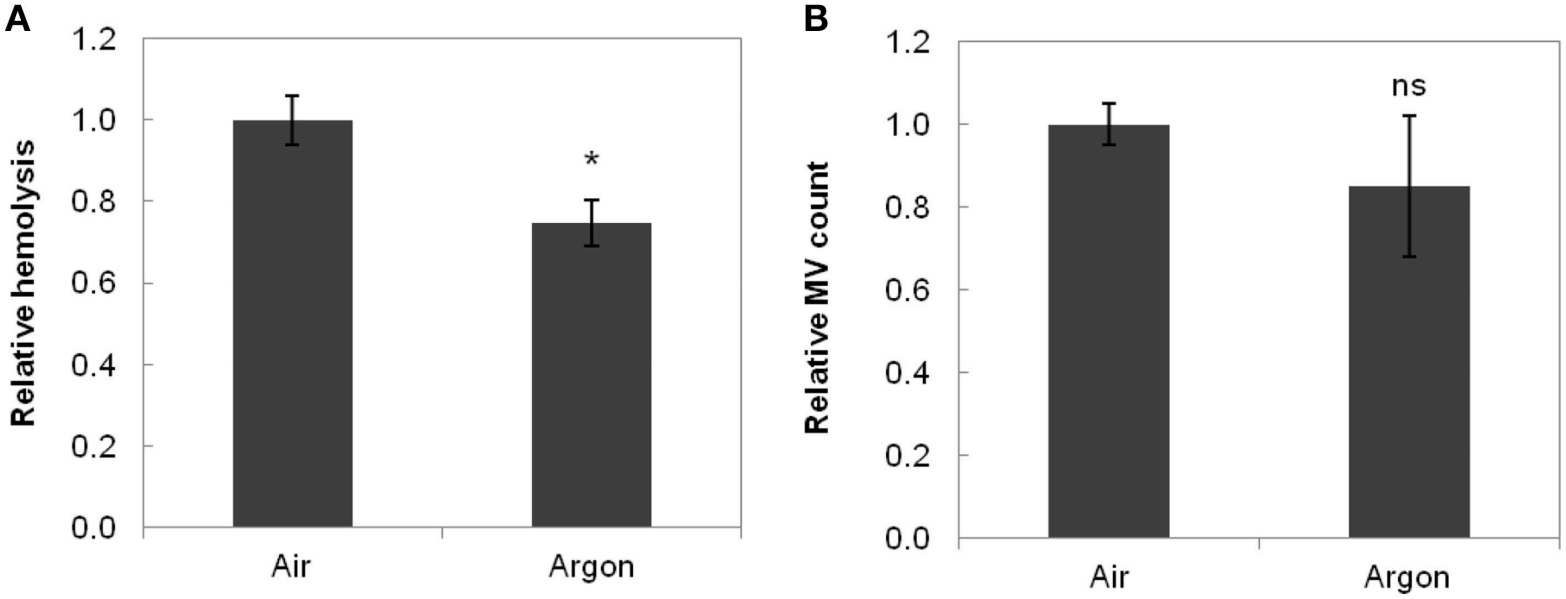
**Hemolysis (A) and MV count (B) in function of storage conditions**. Data were normalized to air conditions before averaging and are the mean relative value ± standard deviation. Hemolysis was significantly lower under argon (^*^p < 0.017, *n* = 3). MV count was lower under argon but not significantly different (*p* = 0.266, *n* = 3). ns stands for non-significant.

The results for hemolysis and MVs are shown in Figure 2. Absolute quantities instead of concentrations were used to take into account the volume difference between the bioreactors. Indeed, medium volume decreased in the bioreactors (~10%, due to evaporation by the gas flow despite the humidifiers), and this decrease was not uniform in the two parallel bioreactors. Thus, the results from the three experiments were pooled after normalization.

Hemolysis [calculated from Equation (2)] reached a value of 1.8 ± 0.5% (air) and 1.4 ± 0.3% (argon) at day 14 (Figure 2A, *p* < 0.01, *n* = 3) for an initial value of 0.12 ± 0.04% indicating a lower hemolysis level under argon. MVs, that increase in number during storage [24,700 ± 6700 MVs/μL at day 14 at RT in both bioreactors against 33,700 ± 13,000 MVs/μL at day 42 under current storage in EC bags (*n* = 3)], also exhibited lower count under argon (Figure 2B).

**Figure 3:**
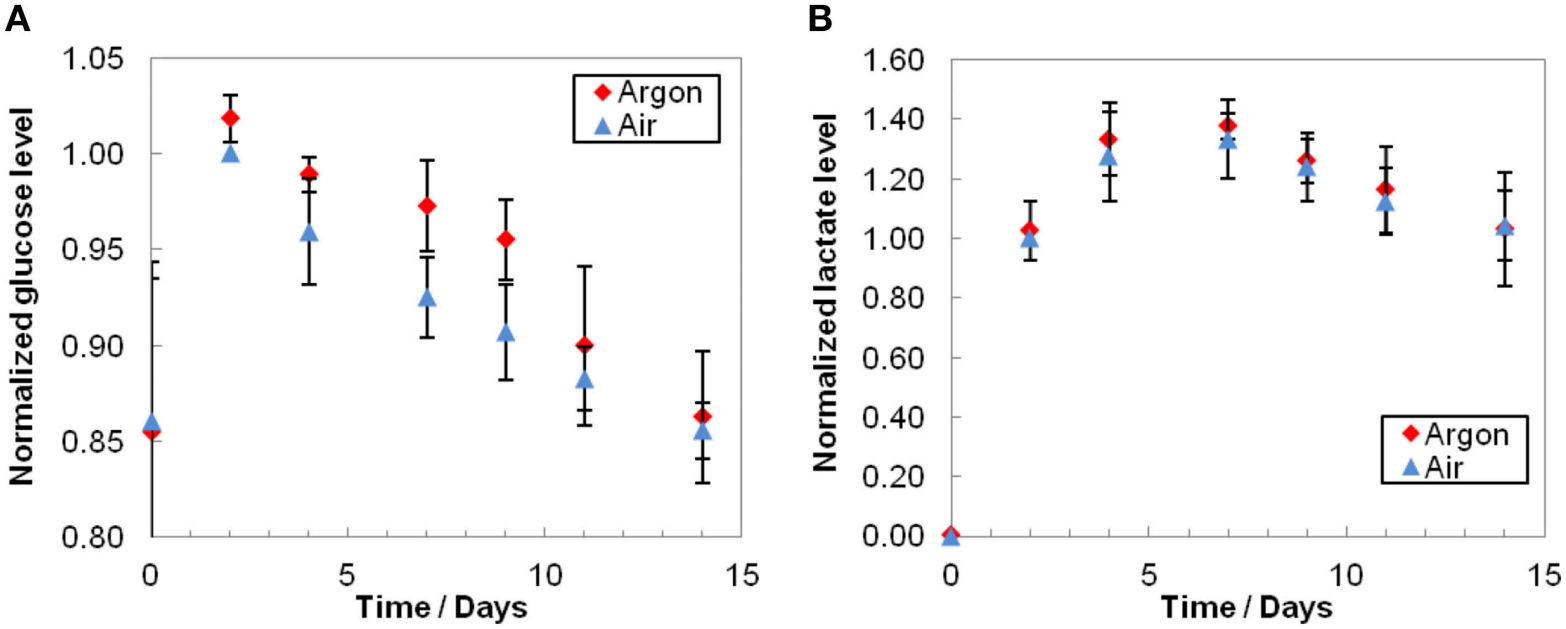
**Extracellular glucose consumption (A) and lactate production (B)**. Average results that were normalized to air day 2 conditions before averaging and are the mean relative value ± standard deviation. As expected, glucose level decreased and lactate level increased during storage. Even though the consumption of glucose was slightly higher under air condition, the difference is not significant (*p* > 0.1, *n* = 3). Lactate productions were equivalent under air or argon (*p* > 0.09, *n* = 3).

The glucose profiles showed a small but constant decrease over time (Figure 3A) and lactate concentrations typically increased until day 7 before slowly diminishing (Figure 3B). Glucose consumption was slightly lower under argon, whereas lactate profiles were equivalent under both conditions. As expected, we observed a substantial inter-donor variability. It should be also noted that extracellular medium samples were taken at day 0 only 1 h after injection of RBCs, i.e., before medium homogenization (RBCs were injected along with the solution contained in the EC, whose composition differs from mSAGM). More than half a day was required to renew the bioreactor volume (residence time, as total volume/flow rate, of 14.9 h), resulting in gaps observed between day 0 and day 2 in glucose concentrations.

### Metabolomics

Fifty three intracellular metabolites were targeted by virtue of their importance in the energy metabolism (e.g., ATP, NADH, glucose) or in the cellular redox balance maintenance (e.g., GSH/GSSG, NADPH) of the RBCs. Other metabolites (e.g., amino acids) were also investigated. As the isomers ribose 5-phosphate/ribulose 5-phosphate/xylose 5-phosphate could not be distinguished by MS, they were grouped. The three types of samples: day 0, day 14 air and day 14 argon, were injected each three times on the LC-MS.

**Figure 4:**
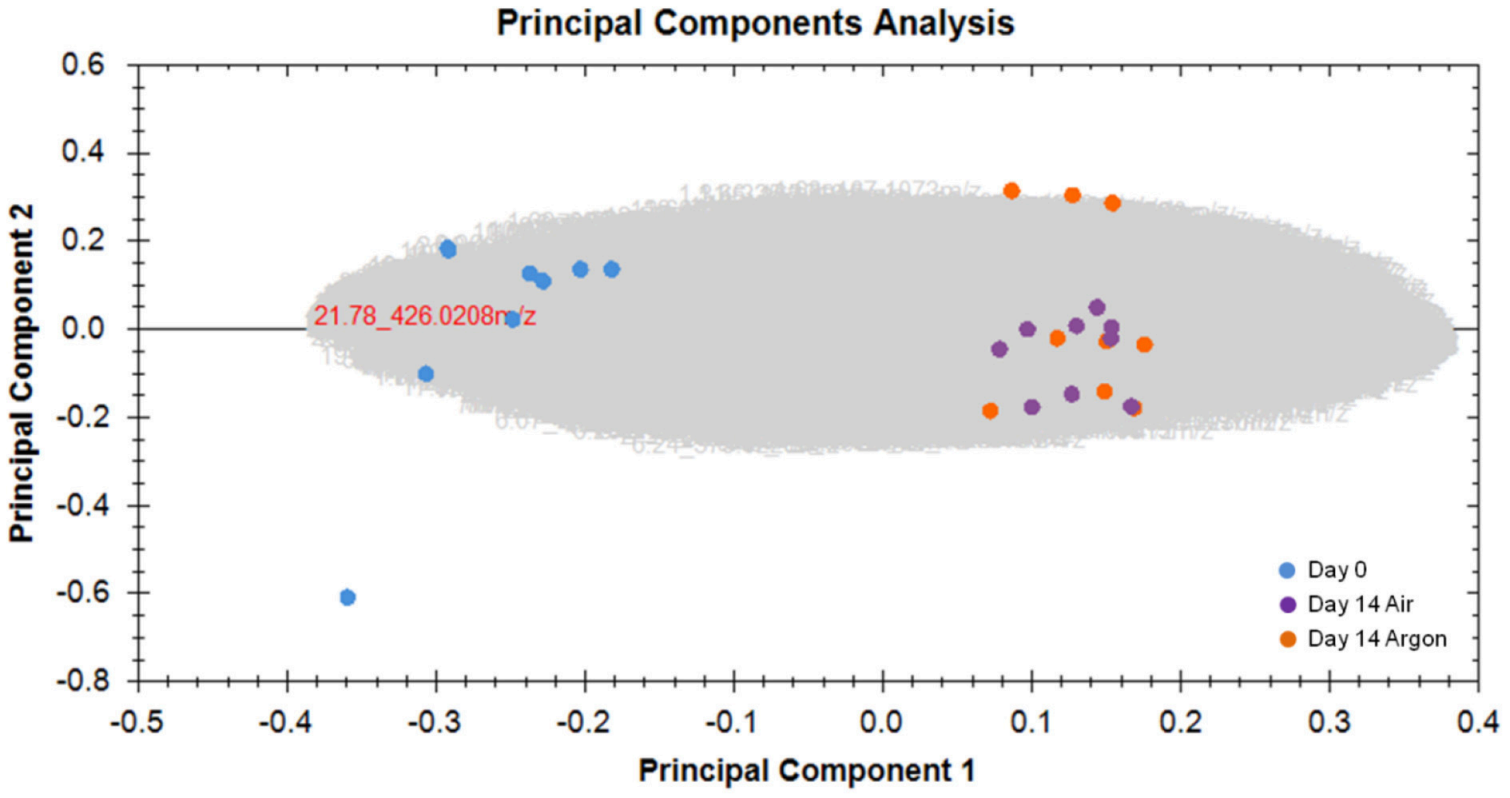
**Principal component analysis of metabolomic data**. RBC aging inside the bioreactors was clearly assessed. Gray cloud represents unidentified compounds. No data filters were used.

PCA highlighted two distinct groups (Figure 4). The effect of aging is clearly observed with a consequential shift in PC1, whereas air and argon conditions at day 14 did not significantly differ.

**Figure 5:**
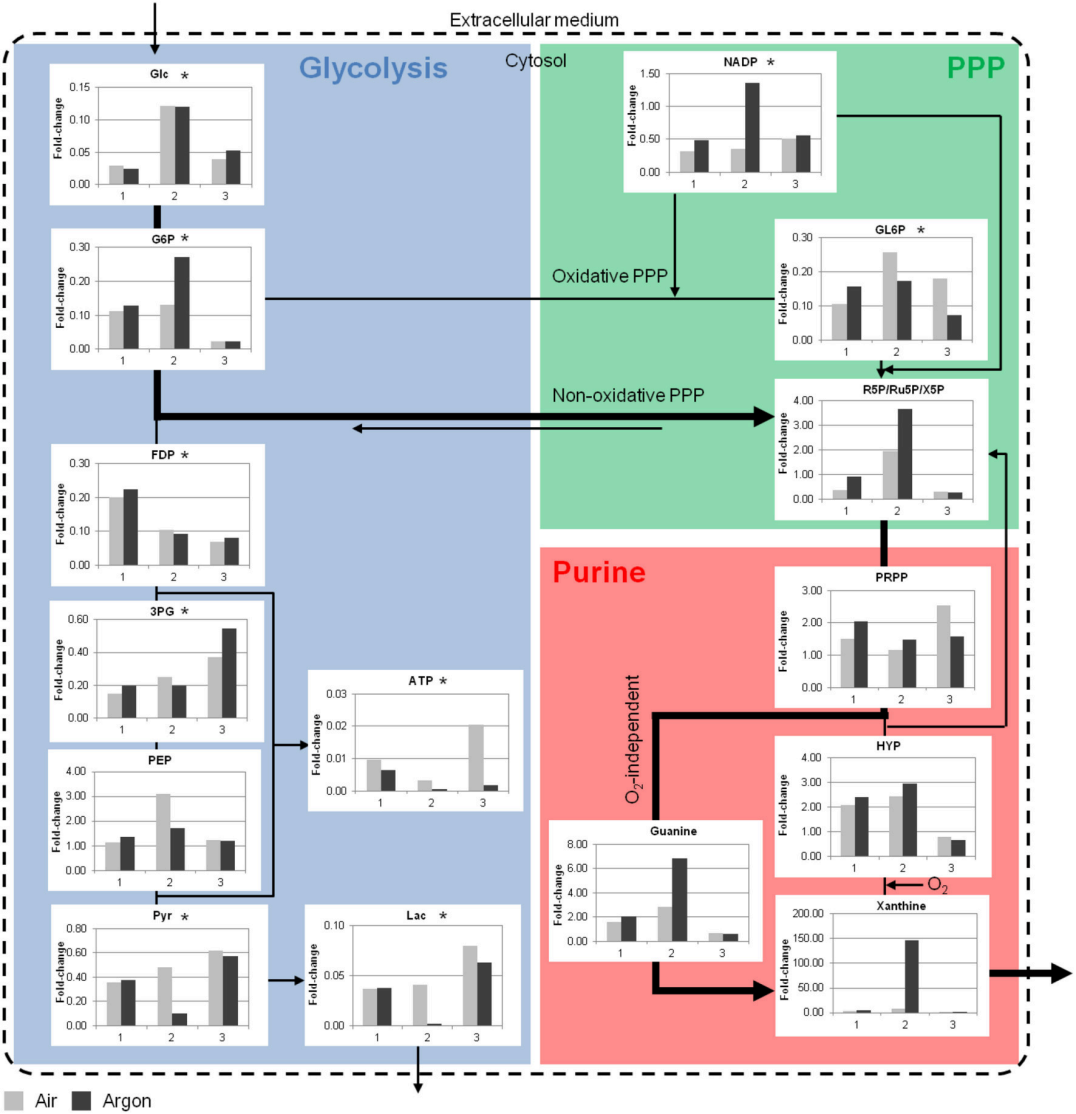
**Simplified overview of key RBC metabolites in the bioreactor storage experiments**. Glycolytic and PPP metabolites were consumed during *ex vivo* aging, whereas accumulations were observed in the purine metabolism. Data are expressed as fold-change compared to day 0. Bold arrows represent the possible shunt from glycolysis to PPP under argon at RT. Glc, glucose; G6P, glucose 6-phosphate; FDP, fructose 1,6-biphosphate; 3PG, 3-phosphoglycerate; PEP, phosphoenolpyruvate; Pyr, pyruvate; Lac, lactate; GL6P, 6-phosphogluconate; R5P, ribose 5-phosphate; PRPP, phosphoribosylpyrophosphate; HYP, hypoxanthine. 1, 2, and 3 are the bioreactor experiment numbers. ^*^p < 0.05 compared to day 0, *n* = 3.

A few key metabolites of the glycolysis, the PPP and the purine metabolism are depicted in Figure 5 (see Supplementary Materials for detailed results). During storage there was a clear depletion of glycolytic metabolites, except for phosphoenolpyruvate that tended to accumulate. The decrease in lactate was consistent with the increase in the extracellular medium. A depletion was also observed in the oxidative PPP and accompanied by an accumulation of metabolites in purine metabolism such as hypoxanthine and xanthine. These accumulations clearly indicated that an important part of the purines were derivate to non-usable products for ATP synthesis. Consequently, ATP was also depleted.

Metabolomic differences between air and argon conditions were extensive. The fast depletion of metabolites in bioreactor storage experiments at RT did not provide clear segregation of the data. Nevertheless, more ATP was consumed (or produced in lower amounts) and less NADP was consumed under argon. In addition to an accumulation of ribose 5-phosphate and purine metabolites, it was hypothesized that non-oxidative PPP was promoted through the conversion of glucose 6-phosphate to ribose 5-phosphate (Figure 5, bold arrow) (Stryer, 1992). This pathway bypasses the production of ATP and NADPH. Ribose 5-phosphate then enters the purine metabolism and it can be either recycled to produce ATP or degraded through xanthine and urate. This last pathway should pass through O_2_-independent reaction in the argon bioreactor since the formation of xanthine from hypoxanthine is catalyzed by xanthine oxidase in an O_2_-dependent manner. Moreover, allantoin, an oxidized product of urate, was less abundant in the RBCs under argon.

It must to be noted that experiments behaved slightly differently each others. For instance, Experiment 2 exhibited higher level of amino-acids under argon than under air (see Supplementary Figure 3); and Experiment 3 showed depletion of purine metabolites. The metabolomics reported here were end-point experiments only and the kinetics during senescence is not reported. Therefore, relative differences may be underestimated since several metabolites were consumed during the bioreactor storage experiments. In addition, donor variability should be considered (Dern et al., 1966; Tzounakas et al., 2015).

## Discussion

### Conceptualization and limitations

The feasibility of maintaining RBCs in a stable small-scale perfusion bioreactor was clearly demonstrated here, and the fluidized-bed was the most suitable design for perfusion since the other tested designs did not allow to reach the requirements. It enabled segregation of cells from the medium without additional filters and back-pressure. With this platform, measurement of on-line parameters and collection of extracellular samples was made possible. The number of cells was minimized to reduce biological sample size and the ECs were 10 times diluted (hematocrit of 3.8 ± 0.9, *n* = 6). SAGM was adapted to insure the same glucose-to-cell ratio (glucose dilution by 10), whereas NaCl (osmolarity and ionic balance), adenine (metabolism maintenance) and mannitol (membrane stabilization) concentrations were kept constant. The main reason for this change was the need to observe a detectable extracellular consumption during the storage, and having a high glucose-to-cell ratio could have influenced results in this regard. Thus, mSAGM was used instead of SAGM.

Moreover, to avoid contamination at this stage of the conceptualization of this small-scale perfusion bioreactor, antibiotics (penicillin and streptomycin) were added, which successfully prevented time-to-time abnormally high hemolysis levels (contamination observed in initially stages of design, data not shown). In a long-term view, the use of antibiotics would be avoided by the employment of completely sterile materials and environment.

Additionally, the efficiency of oxygen-depletion is important. Argon flux may not be sufficient under these conditions to ensure efficient O_2_ depletion (even though bioreactors and reservoirs were under constant gas supply) and could explain the partly muted differences between the gases tested. Therefore, the integration of dissolved oxygen probes (e.g., from PreSens Gmbh, presens.de) is an important step in the evolution of our system for studying aerobic and anaerobic conditions.

### Hemolysis and MVs

Regarding hemolysis and MVs, lower levels were observed under the anaerobic conditions (the difference was significant for hemolysis). This is consistent with our preliminary works and with the hemolysis results found by Yoshida et al. (2007), Yoshida and Shevkoplyas (2010). Since both hemolysis and MV levels increase with storage duration (Hess et al., 2000; Greenwalt, 2006; Yoshida et al., 2007; Rubin et al., 2013; Wagner et al., 2014), they may be considered as the direct consequences of the storage-induced cell damages and lower levels would therefore indicate improved RBC conservation. Under this hypothesis, anaerobic storage would appear as having a positive effect on RBC aging processes, probably by lowering oxidative stress. Alternatively, these hemolysis and microvesiculation phenomena might be protective for the cells, getting rid of deleterious compounds (Willekens et al., 2008; Delobel et al., 2012). MVs contain a level of oxidized proteins that increases with storage duration (Delobel et al., 2012), and therefore microvesiculation may act specifically as a protection against oxidative damage. The decreased levels under argon could point to both the disruption of these protective mechanisms or the reduction of the actual cellular damages.

### Metabolites

The extracellular glucose profiles (Figure 3A) showed a constant decrease consistent with cell consumption. On the other hand, extracellular lactate (Figure 3B), as the end-product of glycolysis, accumulated until reaching a plateau and, and subsequently continued to decrease slightly. A possible explanation may be the presence of lactate dehydrogenase and its activity in the medium at RT, because of higher hemolysis compared to cold storage. However, we cannot exclude a shift of the metabolism that could use pyruvate in other pathways.

The intracellular data are consistent with the literature on *ex vivo* aging (D'Alessandro et al., 2015). The faster depletion observed here is explained by high temperature, as the bioreactors were run at RT. Moreover, in spite of an expected extracellular glucose level, the glycolytic metabolites were impoverished, which is also the case in cold storage (Gevi et al., 2012; Roback et al., 2014). Interestingly, the accumulation of phosphoenolpyruvate and decrease of pyruvate during bioreactor storage could be explained by a reduced activity of pyruvate kinase. Delobel et al. reported that this enzyme was carbonylated during cold storage of RBCs and that the absence of it at the end of the storage could be explained by protein relocation or elimination through microvesiculation (Delobel et al., 2012, 2016).

Comparing aerobic vs. anaerobic storage, the extracellular glucose consumption appeared to be lower under argon (though not significant), whereas lactate production was equivalent. Moreover, the end-point metabolomics does not allow for distinguishing clear effects since several metabolites were already depleted. These results are not consistent with the observations of Yoshida's and Zolla's groups (Yoshida et al., 2007, 2008; Dumont et al., 2009, 2016; Yoshida and Shevkoplyas, 2010; D'Alessandro et al., 2013, 2015). They indeed reported both higher glucose consumption and higher lactate production under oxygen-depletion, as well as a general increase in glycolysis as O_2_ partly inhibits it. It has been proposed that oxygen depletion forces the hemoglobin contained in RBCs into its deoxygenated state (Lewis et al., 2009; Castagnola et al., 2010). It results in (i) the increased hemoglobin affinity for protons, leading to higher pH, and therefore increased phosphofructokinase (the glycolysis rate-limiting enzyme) activity; (ii) increased hemoglobin affinity for 2,3-DPG and ATP, and thus reduced inhibition of phosphofructokinase; (iii) increased hemoglobin affinity for the band 3 protein causing release of phosphofructokinase and other glycolytic enzymes. Hence, hypoxia induces a shift from the PPP to the glycolysis (Kinoshita et al., 2007; Rogers et al., 2009; Dumont et al., 2016). Under these conditions, the recycling capacity of antioxidants like glutathione is lowered because the PPP does not produce enough NADPH. A shunt to the non-oxidative PPP (known in case of ribose 5-phosphate need and not NADPH, Stryer, 1992) could be one explanation of the differences observed under our bioreactor working at RT (Figure 5).

These interpretations go beyond the aim of this project and will require a more advanced system and replicates. However, they point out the role of such bioreactors and their improvements. Monitoring and controlling the pH appear thus important. The integration of pH (and potassium) electrodes and O_2_ probes are already planned for the future development, and will bring insights on storage lesions.

## Conclusions

A small-scale perfusion bioreactor of RBCs was designed and has been developed to limit the quantity of cells used, to constantly segregate RBCs from the medium without filters and to enable the setup of different storage or environmental conditions. The development reached so far operates at a flow rate of 8.5 μL/min, includes gas supply and runs over a 14-day period at RT. Moreover, the design allows for easy sampling of the extracellular medium and cells thanks to the fluidized bed system. Remarkably, it enables us to change the bioreactors medium in a dynamic manner (since it is permanently separated from cells). Beyond the cell segregation and the simplified monitoring of extracellular compartment, the principle was proven by studying hemolysis, microvesiculation, and extracellular and intracellular metabolites. The targeted metabolomic approach clearly reported the depletion of glycolytic, PPP and energy metabolites. Even though the RBC metabolism under aerobic and anaerobic conditions has to be further studied kinetically, this approach appears more than suitable to study RBCs (Prudent et al., 2014). It is able to challenge RBCs and mimic their pathways in response to different stimuli. For instance, the combination of antioxidants and anaerobic conditions could be investigated in order to take advantage of them (D'Alessandro et al., 2013; Pallotta et al., 2014).

Further bioreactor development will employ new probes for on-line monitoring and consider other gases (or gas composition, Dumont et al., 2016) and AS. An O_2_-probe to better control depletion efficiency is currently under implementation. This small-scale perfusion bioreactor potentially gives access to a high number of difficult-to-obtain parameters (on-line or off-line) and could be coupled to other tools such as NMR (Gmati et al., 2005). It is of high importance to deeper understand this complex cell and the signaling involved under various conditions.

This fundamental approach is not only of importance in transfusion medicine since such bioreactor enable the comparison of different AS formulation and metabolic supply, but also for understanding RBC senescence (in whole blood, for instance) including different metabolic pathways, protein modification, microvesiculation, defense mechanisms and so forth. The goal of such a system is to provide innovative strategies to reconsider RBC storage. This development gives new insight in the way of handling RBCs in an open system.

## Author contributions

MP initiated the project and the conception, reviewed improvements, analyzed data and wrote the article. SH made the first developments and designed the fluidized-bed bioreactor. AR improved the bioreactor and fixed hemolysis issues. NP and FS carried out experiments and improved the setup and quantification of metabolites. All of them contributed to the writing of the manuscript. LM and BR ran metabolomic experiments. MA ran statistical analysis and reviewed data. JT and NL reviewed the data and the manuscript. All the authors read and approved the final manuscript.

### Conflict of interest statement

The authors declare that the research was conducted in the absence of any commercial or financial relationships that could be construed as a potential conflict of interest. The reviewer (MA) and Handling Editor declared their shared affiliation, and the Handling Editor states that the process nevertheless met the standards of a fair and objective review.

## Abbreviations

2, 3-DPG: 2,3-bisphosphoglycerate
AS: additive solution
ATP: adenosine triphosphate
ECs: erythrocyte concentrates
GSH: glutathione
Hb: hemoglobin
LC: liquid chromatography
RBCs: red blood cells
MS: mass spectrometry
mSAGM: modified SAGM
MVs: microvesicles
PCA: Principal component analysis
PPP: pentose phosphate pathway
RT: room temperature
SAGM: saline-adenine-glucose-mannitol.
