# Supplementary material for: Small-Scale Perfusion Bioreactor of Red Blood Cells for Dynamic Studies of Cellular Pathways: Proof-of-Concept
Source: Front Mol Biosci. 2016 Mar 30;3:11. doi: 10.3389/fmolb.2016.00011 (PMC4812044; doi:10.3389/fmolb.2016.00011)
Supplement: Supplementary file 1 [file DataSheet1.docx]

Supplementary Material

Small-scale perfusion bioreactor of red blood cells for dynamic studies of cellular pathways: proof-of-concept

Michel Prudent1*, Frédéric Stauber1, Alexis Rapin1, Sonia Hallen1, Nicole Pham1, Mélanie Abonnenc1, Laure Marvin1,2, Bertrand Rochat2, Jean-Daniel Tissot1, and Niels Lion1

*** Correspondence:** Corresponding Author: michel.prudent@itransfusion.ch

# Designs of tested bioreactors

# Before ending-up with the design presented in this manuscript, different designs were considered to fulfill the requirements, i.e. segregated cells from the medium, provide additive solutions and enable sampling (see Supplementary Figure 1).


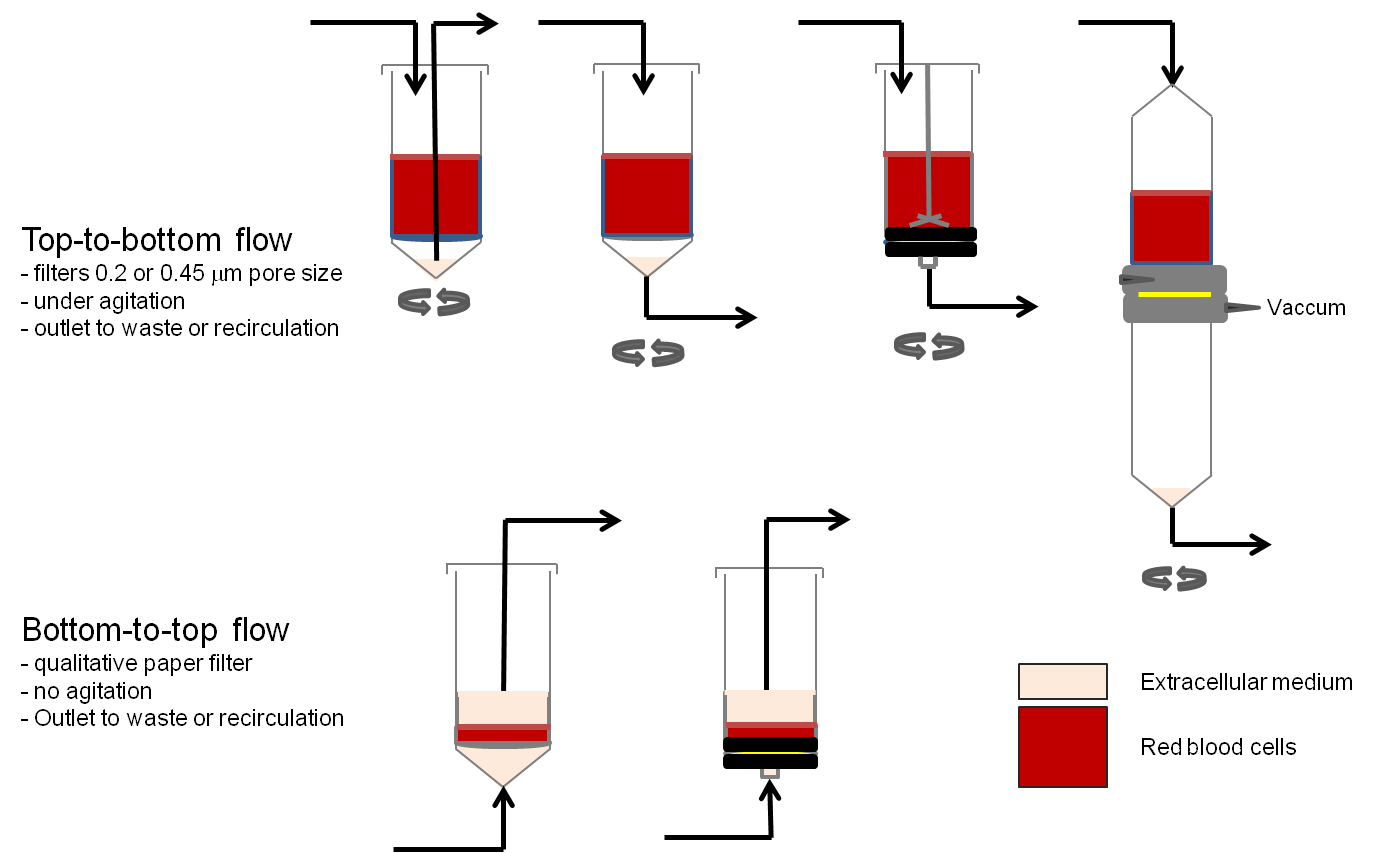


**Supplementary Figure 1**. **Examples of tested bioreactors.** Only main chambers were depicted and reservoirs were not drawn. Outlets can go to waste or go back to the main chambers for medium recirculation.

# The top-to-botttom flow designs required to agitate the bioreactors to homogenize red blood cells (RBCs), and the use of a small pore size filter (< 1 μm) to filter the RBCs able to pass through micrometer pores because of their high deformability. These bioreactors proved to be unsuccessful. They showed leaks in the filters, allowing RBCs to be circulated through the pump and increased hemolysis. Moreover, more cells were lyzed due to increase pressure drops across mirco-pore filters.

The perfusion based on fluidization of RBCs (bottom-to-top flow) does not required micro-pore filter since the segregation of RBCs was successfully achieved thank to the balance between sedimentation and fluid flow force. No agitation was required in this configuration.

# Effect of albumin concentration on hemolysis

# RBCs from an 8-day old erythrocyte concentrate (EC) were washed 4 times in 0.9% NaCl (centrifugation at 2000g, 10 min, 4°C). RBCs were diluted at a hematocrit of 0.42 in SAGM containing 0 to 40 g/L of bovine serum albumin (BSA) (total volume of 2 mL in a 15-mL tubes). Tubes were closed and vents were inserted in the cap (18G syringe with a 0.2 μm pore filter). RBCs were stored 7 days at room temperature (RT). Hemolysis was measured as described in section 2.4 of the main manuscript in tubes as well as in the EC as control.


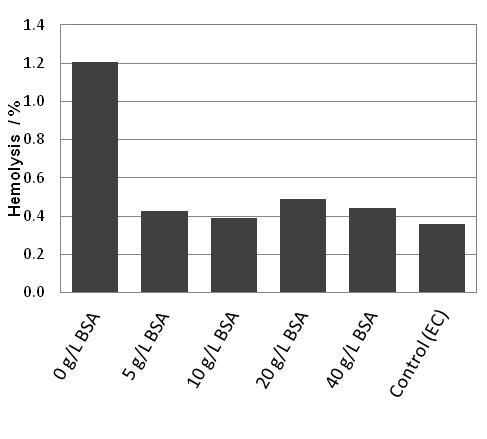


**Supplementary Figure 2**. **Hemolysis in function of albumin concentration.**

Over a 7-day period at RT, the absence of albumin induced an approximately 3 fold increase compared to storage in presence of 5 g/L BSA (0.4% hemolysis in presence of 5 g/L BSA vs 1.2% without BSA). The level of hemolysis within the 15-mL tubes in presence of BSA was equivalent in the EC (see Supplementary Figure 2).

# Metabolomic data


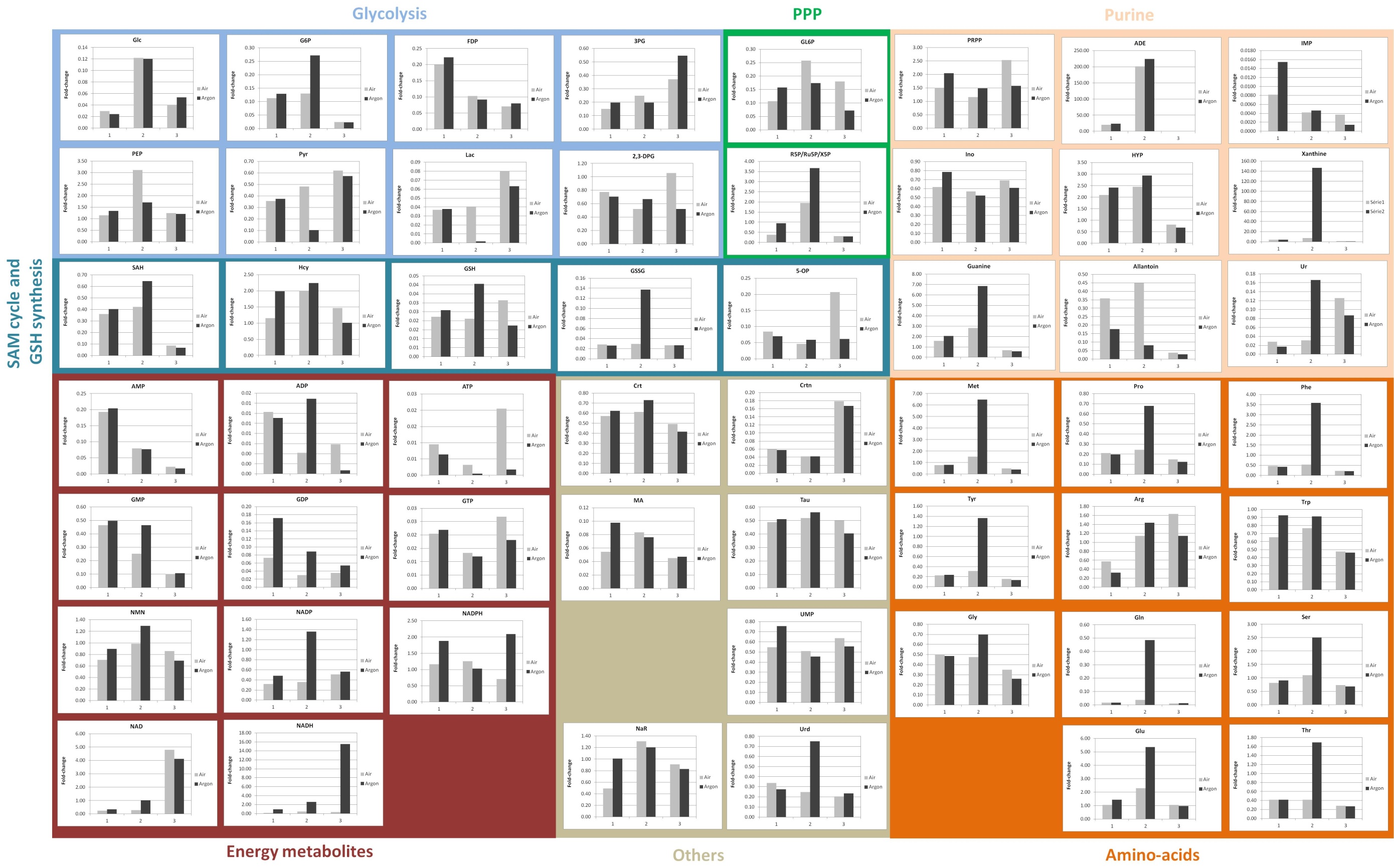


**Supplementary Figure 3**. **Targeted metabolites in different pathways.** Relative quantitation is expressed as fold-change compared to day 0. 1, 2 and 3 are the bioreactor experiment numbers. PPP: pentose phosphate pathway, SAM: S-adenosylmethionine, GSH: glutathione.
